# Novel Mechanisms Revealed in the Trachea Transcriptome of Resistant and Susceptible Chicken Lines following Infection with Newcastle Disease Virus

**DOI:** 10.1128/CVI.00027-17

**Published:** 2017-05-05

**Authors:** Melissa S. Deist, Rodrigo A. Gallardo, David A. Bunn, Terra R. Kelly, Jack C. M. Dekkers, Huaijun Zhou, Susan J. Lamont

**Affiliations:** a Department of Animal Science, Iowa State University, Ames, Iowa, USA; b Department of Population Health and Reproduction, School of Veterinary Medicine, University of California, Davis, California, USA; c Department of Animal Science, University of California, Davis, California, USA; d One Health Institute, University of California, Davis, California, USA; IIS/LAD/NIAID/NIH

**Keywords:** Newcastle disease virus, RNA-seq, trachea, chicken, collagen, eIF2 signaling

## Abstract

Newcastle disease virus (NDV) has a devastating impact on poultry production in developing countries. This study examined the transcriptome of tracheal epithelial cells from two inbred chicken lines that differ in NDV susceptibility after challenge with a high-titer inoculum of lentogenic NDV. The Fayoumi line had a significantly lower NDV load postchallenge than the Leghorn line, demonstrating the Fayoumi line's classification as a relatively NDV-resistant breed. Examination of the trachea transcriptome showed a large increase in immune cell infiltration in the trachea in both lines at all times postinfection. The pathways conserved across lines and at all three time points postinfection included iCOS-iCOSL signaling in T helper cells, NF-κB signaling, the role of nuclear factor of activated T cells in the regulation of the immune response, calcium-induced T lymphocyte apoptosis, phospholipase C signaling, and CD28 signaling in T helper cells. Although shared pathways were seen in the Fayoumi and Leghorn lines, each line showed unique responses as well. The downregulation of collagen and the activation of eukaryotic translation initiation factor 2 signaling in the Fayoumis relative to the Leghorns at 2 days postinfection may contribute to the resistance phenotype seen in the Fayoumis. This study provides a further understanding of host-pathogen interactions which could improve vaccine efficacy and, in combination with genome-wide association studies, has the potential to advance strategies for breeding chickens with enhanced resistance to NDV.

## INTRODUCTION

Newcastle disease is caused by virulent strains of Newcastle disease virus (NDV), a single-stranded, negative-sense, nonsegmented paramyxovirus that negatively impacts poultry meat and egg production throughout the world. In developing countries, vaccination is sometimes not a viable option, due to a lack of infrastructure (cold chain and transportation), hampering the availability of vaccines; a lack of technically trained personnel; the cost of the vaccine; and the need for readministration. Other solutions must be found in order to control this disease (1). In these countries, virulent NDV can cause mortality at rates as high as 80% among village flocks (2). Ameliorating the negative impacts of NDV would reduce hunger, strengthen food security, alleviate poverty, and, since chickens are traditionally a woman's responsibility, promote gender equality in developing countries (3).

In developed countries, NDV also has the potential to cause severe damage to the poultry industry from direct and indirect losses due to trade embargoes and restrictions during outbreaks. Hitchner (4) refers to NDV as a “sleeping giant” that requires close monitoring because the large range of clinical signs of infection with NDV and the genomic diversity of NDV make NDV detection and diagnosis challenging (5, 6), the worldwide distribution increases the potential danger of NDV (5), and, although they are uncommon, viruses of low virulence have the ability to mutate and increase in virulence (7).

The range of clinical signs in chickens infected with NDV varies depending on the route of infection, environmental factors, host immunity, and the strain of the virus (8). The strains of NDV are divided into three major categories ranging from low to high virulence: lentogenic, mesogenic, and velogenic (8). The intracerebral pathogenicity index (ICPI) is used to differentiate and classify NDV according to its virulence (8), which is largely determined by the F protein cleavage site (9). Although the strains differ in their virulence, all belong to the same serotype, thus enabling the use of lentogenic viruses as vaccines for protection against the more virulent strains. Although infections by lentogenic strains are rare, clinical signs associated with infections by lentogenic strains are nonspecific and include ruffled feathers, anorexia, decreased egg production, and respiratory infection in birds with a compromised immune system (5, 6, 10).

Understanding the host response to NDV is necessary in order to generate improved solutions to combat this devastating disease. Examination of host mRNA expression after challenge provides insight into the host-pathogen interaction. Previous studies have compared gene expression levels in response to infection with multiple NDV strains and in host tissues measured from quantitative PCR (qPCR) and microarray data and reported different cytokine expression levels dependent on the strain causing the infection (11–15). Gene expression analyses performed on tracheal epithelial cells following NDV infection *in vitro* showed a strong innate immune response (16). The trachea is one of the first tissues that NDV encounters when it is transmitted by the aerosol route and a critical site of the host-pathogen interaction. All strains of NDV are able to replicate in the epithelial cells of the trachea (17), and challenges with either lentogenic or velogenic strains resulted in comparable viral titers in the trachea (18).

Although lentogenic NDV generally does not cause severe clinical signs, gene expression changes due to inflammation, tissue destruction, cell proliferation, and tissue remodeling are expected in the trachea after intranasal inoculation (19). Aerosol delivery can result in deciliation, congestion, goblet cell hyperplasia, edema, and infiltration of heterophils, lymphocytes, and plasma cells in the tracheal mucosa (20, 21). If one is seeking mechanisms of host resistance, the trachea is an ideal tissue to examine, as this is one of the initial sites of interaction with the virus.

Host genetics may play a large role in the host-pathogen interaction. Previous studies have shown that resistance to NDV has a genetic basis (22–25). In the current study, two diverse, inbred chicken lines, the Fayoumi and Leghorn lines, were utilized to identify genetic mechanisms of resistance to NDV. Several studies have compared the immune responses of these two lines. Chickens of the Fayoumi line were found to be relatively resistant to Salmonella, Eimeria, Marek's disease, and avian influenza virus (AIV) compared to those of the Leghorn line (26–29). Bone marrow-derived dendritic cells from these two lines showed increased phagocytic ability, nitric oxide production, and major histocompatibility complex (MHC) class II surface expression (30). Here the Fayoumi and Leghorn lines were used as a discovery platform to identify mechanisms of resistance to NDV. We hypothesized that the Fayoumis are more resistant to NDV, as shown by a lower viral load and a higher antibody titer. The term “resistance,” as used in the current study, is defined as the ability of the host to interfere with the pathogen life cycle (31). The term resistance in this context is not absolute; therefore, the host may still become infected and succumb to infections caused by virulent NDV strains. Comparison of the two lines' responses to NDV may designate genes/pathways associated with the resistance of the Fayoumi line. The information generated by this study will be beneficial for vaccine development and other control strategies.

## RESULTS

### The Fayoumi line's resistance phenotype was upheld by the NDV viral load, antibody quantification measurements, and sequence data.

**FIG 1:**
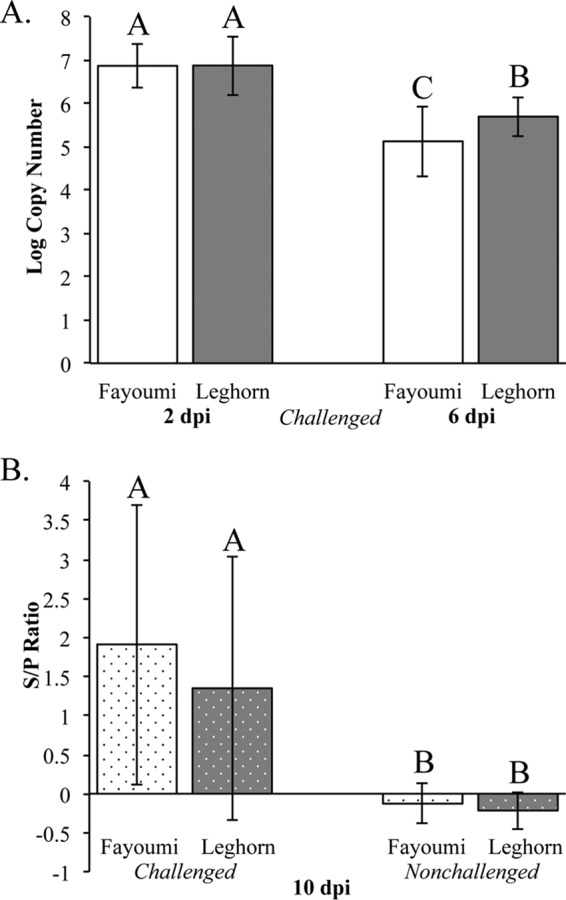
Viral load and antibody quantification by genetic line and day postinfection. (A) The viral load is shown as the least-square means of the log copy number, measured by qPCR, of the Fayoumis (white) and Leghorns (gray). Error bars represent standard deviations. A Student *t* test was used to derive the connecting letters report, in which the values for bars that are not labeled with the same letter are significantly different (*P* < 0.05). At 2 dpi, data are for 21 Fayoumi and 28 Leghorn chickens, and at 6 dpi, data are for 12 Fayoumi and 20 Leghorn chickens (total *n* = 81). (B) The antibody titer is displayed as the least-square means of the S/P ratio in the Fayoumis (white) and Leghorns (gray) at 10 dpi, and the error bars represent standard deviations. The connecting letters report was generated by Student's *t* test. At 10 dpi, data are for 8 challenged Fayoumi chickens, 13 challenged Leghorn chickens, 6 nonchallenged Fayoumi chickens, and 8 nonchallenged Leghorn chickens (total *n* = 35).

To examine the effects of an NDV challenge on the two inbred lines with different resistance, the challenged Fayoumi and Leghorn chickens were inoculated with the La Sota NDV strain, while the nonchallenged chickens were given saline solution as a mock infection. Lachrymal fluid was collected from each chicken prior to infection, at 2 days postinfection (dpi), and at 6 dpi for viral quantification. No NDV was detected in any of the birds prior to challenge or in any of the nonchallenged birds at all times (data not shown). In the challenged birds, the viral load significantly decreased from 2 to 6 dpi (*P* < 0.0001), and the line had a significant effect on the viral load (*P* = 0.045) (Fig. 1). At 6 dpi, the Fayoumis had significantly less virus than the Leghorns (*P* = 0.0122), suggesting that the Fayoumis may have cleared the virus more quickly (Fig. 1). There was no correlation (*r* = −0.0097) between each individual's viral load at 2 and 6 dpi, which was in agreement with the findings of previous studies (19).

Serum from blood collected at 10 dpi was used to quantify NDV-specific antibody via enzyme-linked immunosorbent assay (ELISA). The ELISA sample-to-positive (S/P) ratios at 10 dpi were significantly different (*P* = 0.0007) between the challenged and nonchallenged birds. Due to the large variation in NDV antibody levels, there was no significant difference between the lines within the same treatment category. However, the challenged Fayoumis produced more antibodies, on average (*P* = 0.367), than the challenged Leghorns (Fig. 1).

**FIG 2:**
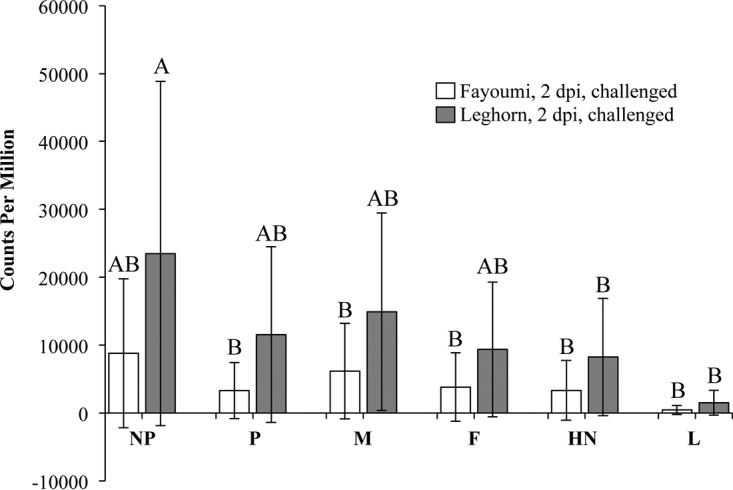
Counts per million aligned to each viral gene in the challenged Fayoumi and Leghorns at 2 dpi. The NDV genes are listed in the order nucleoprotein (NP), phosphoprotein (P), matrix protein (M), fusion protein (F), hemagglutinin-neuraminidase (HN), and large polymerase protein (L). The least-square means of the counts per million are shown for the challenged Fayoumis (*n* = 4) and Leghorns (*n* = 5). Error bars represent standard deviations of the means. The connecting letters report was generated with a Student *t* test.

Sequence reads that did not map to the chicken genome were analyzed to determine if any mapped to genes in the NDV genome (Fig. 2). Viral transcripts were detectable in the challenged birds only at 2 dpi. The main effect of line had a statistically significant impact (*P* = 0.0264) on the viral transcripts of the challenged Fayoumis and Leghorns at 2 dpi (Fig. 2). As expected, the counts per million (cpm) appeared to be higher for genes at the beginning of the virus genome in both lines.

### Effects of NDV challenge on the trachea transcriptome.

Epithelial cells were peeled from the trachea of challenged and nonchallenged birds from both lines at all three times and used for RNA isolation, cDNA library construction, and sequencing. The tracheal epithelium sequencing results were similar across treatment groups, and therefore, we did not expect biases due to differences in the number of reads, mapping percentage, or transcriptome coverage percentage among the treatment groups (Table 1). On average, approximately 2 million reads were removed from each sample in the filtering process, nearly 90% of the filtered reads were mappable, and about 75% of the Galgal4 transcripts had at least one mapped read (Table 1).

**TABLE 1:** Sequence processing summary averages^*a*^

Group	Avg no. of reads		Avg mapping %^*b*^	Avg transcriptome coverage %^*c*^
	Prefiltering	Postfiltering		
Challenged	14,351,100	11,019,846	89.7	76.0
Nonchallenged	14,478,105	11,909,690	89.8	75.7
Fayoumi	13,487,623	11,122,267	89.8	76.0
Leghorn	14,313,716	11,770,191	89.8	75.7
Overall	13,900,670	11,446,229	89.8	75.8

a Averages were taken across four treatment groups.

b The mapping percentage was calculated as the number of reads mapped to the reference genome divided by the number of reads postfiltering.

c Transcriptome coverage percentage was calculated as the number of transcripts with at least one mapped read divided by the total number of transcripts.

**FIG 3:**
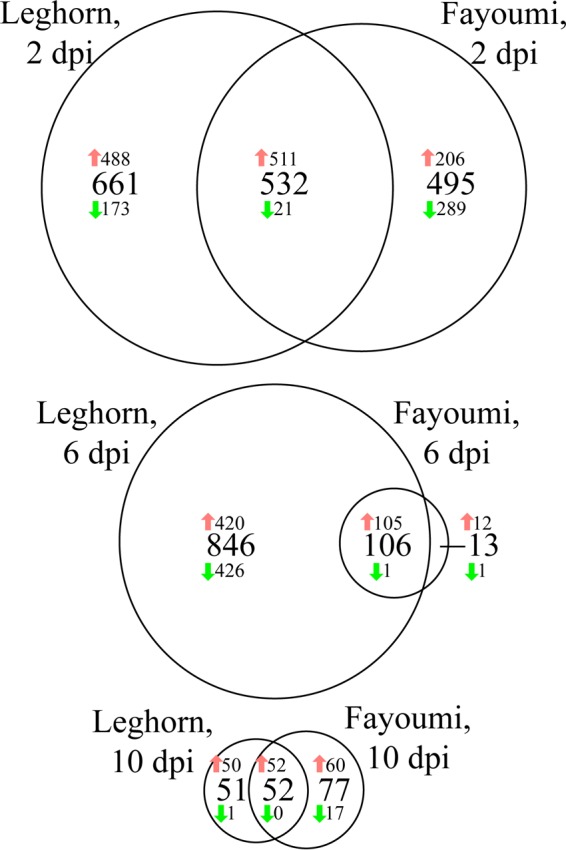
Numbers of differentially expressed genes between challenged and nonchallenged birds within each genetic line at three time points. An FDR of <0.05 was used to classify genes as differentially expressed. Within each portion of the Venn diagram, the numbers of genes that were upregulated and downregulated in the challenged birds relative to the nonchallenged birds are denoted with red and green arrows, respectively.

To compare the host responses to NDV, the responses of challenged and nonchallenged birds within each line were compared at each time point in the experiment. These comparisons are referred to as the six major contrasts. As time progressed, there were fewer differentially expressed (DE) genes (DEG) between the challenged and nonchallenged birds in both lines (Fig. 3). Overall, more genes were upregulated than downregulated (Fig. 3). Differentially expressed genes that were shared between lines are likely crucial to the response to NDV for all chickens, whereas genes uniquely identified as differentially expressed in the Leghorn or the Fayoumi chickens may be related to the susceptibility and resistance, respectively, of the chickens. The gene for protein kinase C beta (PRKCβ) was the only gene that was uniquely identified to be DE only in the Fayoumis at all three time points. The large difference in the numbers of DEG at 6 dpi in the Fayoumi and Leghorn lines (Fig. 3) suggests that the Fayoumi chickens recovered more quickly from the virus infection, resulting in fewer differences between the challenged and nonchallenged birds.

### Challenge with NDV stimulated the infiltration and migration of immune cells.

**FIG 4:**
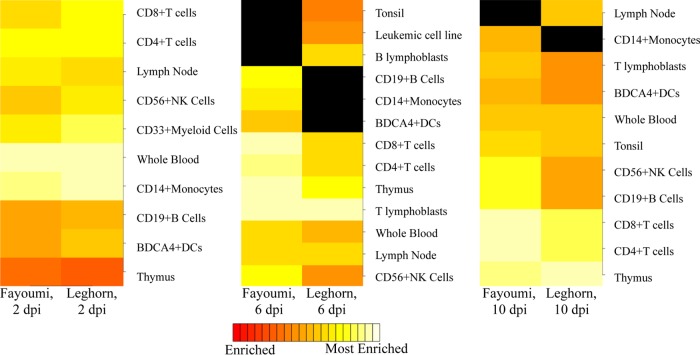
Cell type enrichment analysis predicts enriched cell types from DEG between challenged and nonchallenged birds within each line at each time. The top 10 most significantly enriched cell types (listed to the right of each heat map) range from enriched (red) to most enriched (white) in either the Fayoumis or Leghorns, grouped by time. A black fill indicates a cell type that does not fall into the top 10 enriched cell types of one line but does in the other. Abbreviations: DCs, dendritic cells; Leukemic cell line, leukemia promyelotic HL-60 cells; B lymphoblasts, Burkitt's lymphoma (Daudi); T lymphoblasts, leukemia lymphoblastic (MOLT-4) cells.

The DEG from the challenged versus nonchallenged birds at each time point and within each line were used for cell type enrichment analysis. At 2 dpi, the top cell types enriched were similar for the two lines (Fig. 4); whole-blood cells were the most enriched cell type for both lines. At 6 dpi, the lines were the most dissimilar, which corresponds to the difference in DEG numbers (Fig. 3 and 4). The enrichment of cancer-type cell lines at 6 dpi may have been a result of high levels of cell proliferation. Overall, the results suggested a conserved response between the two lines and a strong enrichment of immune-related cells after challenge at all time points but no clear shifts from innate to adaptive immune cell types over time.

**FIG 5:**
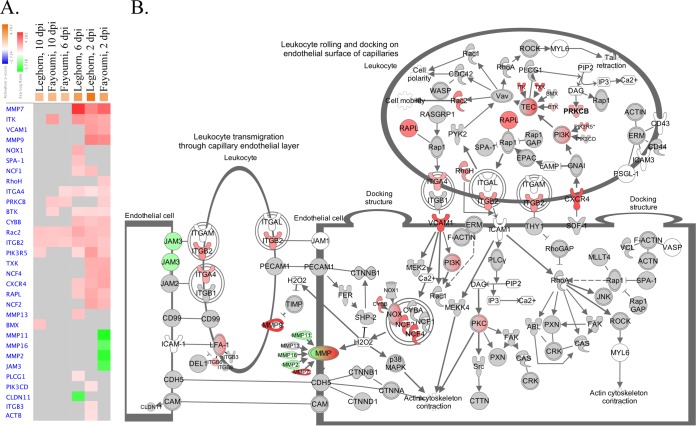
Leukocyte extravasation signaling pathway. For all genes, the absolute LFC was >1 and the FDR was <0.05. (A) Gene expression heat map of genes in the leukocyte extravasation signaling pathway generated by contrasting the challenged and nonchallenged birds within each time and line. The contrasts, listed atop each column, were clustered by similarity on the basis of genes (blue text) that were upregulated (red), that were downregulated (green), and that had no change (gray). Genes that were not DE in any of the comparisons were removed from the graph. This pathway was activated in all contrasts (orange), as predicted by IPA. (B) The leukocyte extravasation signaling pathway in the Fayoumis at 2 dpi. Genes were upregulated (red), downregulated (green), or not DE (gray) in the challenged relative to the nonchallenged chickens. Genes in white were not found in the data set because they were removed during normalization, unrecognized by IPA, or not found in the chicken genome. The shapes of the genes represent their function. The genes within a parent node that were DE in any of the six contrasts are shown individually next to the parental node and are denoted with smaller molecules and labels.

A major pathway predicted by Ingenuity pathway analysis (IPA) to be activated in all six major contrasts was the leukocyte extravasation signaling pathway. Figure 5A shows the genes in this pathway that were significantly up- or downregulated within each contrast. On the basis of the expression levels of the genes in this pathway (Fig. 5B), the molecule activity prediction function of IPA predicted, for each contrast, activation of cell mobility and activation of actin cytoskeleton contraction; the IPA output placed more confidence in the prediction of the latter in the Fayoumis than the Leghorns at all time points (not shown).

### Temporal changes influenced the pathways that were activated or inhibited in each line.

**FIG 6:**
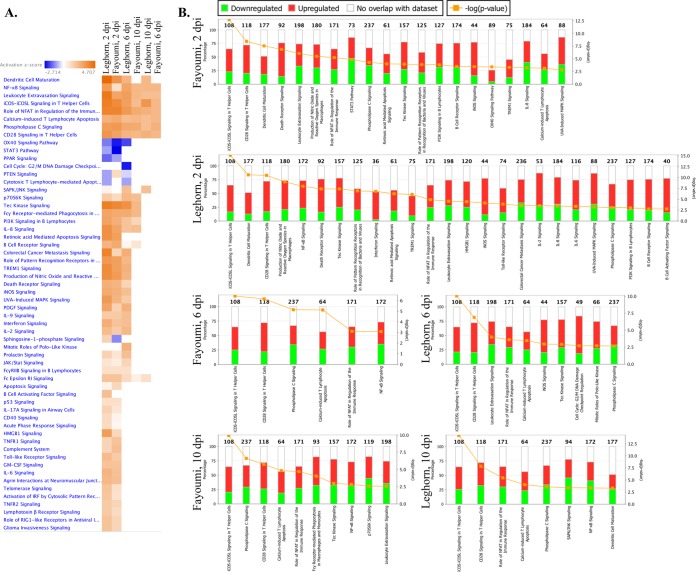
Pathways predicted to be activated or inhibited in response to NDV in the six major contrasts. For all genes, the absolute LFC was >1 and the FDR was <0.05. (A) The heat map, generated by Qiagen IPA software, shows pathways that were either activated (orange), inhibited (blue), or unchanged (white) when the findings for the challenged and nonchallenged birds within each line were contrasted at the three separate time points. The pathways are sorted by hierarchical clustering, and the contrasts (columns) are clustered by similarity. Pathways are filtered by a −log(*P* value) of >2.5 and a Z-score of >1. The complete names of the pathways for entries with ellipses are provided in panel B. (B) The top pathways [those with a −log(*P* value) of >2.5 and a Z-score of >2] for each contrast are shown. The total number of genes in each pathway is found at the top of each bar, which is divided into genes that are downregulated, upregulated, and not DE in the gene data set according to the percentage of the total number of genes in each pathway. The orange lines represent the −log(*P* value) of each pathway. NFAT, nuclear factor of activated T cells; SAPK, stress-activated protein kinase; JNK, Jun N-terminal protein kinase; PI3K, phosphatidylinositol 3-kinase; iNOS, inducible nitric oxide synthase; MAPK, mitogen-activated protein kinase; PDGF, platelet-derived growth factor; IL-9, interleukin-9; GM-CSF, granulocyte-macrophage colony-stimulating factor.

The top canonical pathways predicted by IPA are shown in Fig. 6 for each of the six major contrasts. The six major contrasts were clustered by similarity, which was determined by the resemblance of the activation or inhibition scores of all the pathways. The clustering grouped the major contrasts by dpi, with the Fayoumis at 6 dpi being the outlier (Fig. 6). At 6 dpi there were only 119 DEG for the Fayoumis to populate the pathways, making it more difficult to obtain significance for activation or inhibition of a pathway. Several key pathways were significantly activated at all time points in both lines: iCOS-iCOSL signaling in T helper cells, NF-κB signaling, the role of the nuclear factor of activated T cells in the regulation of the immune response, calcium-induced T lymphocyte apoptosis, phospholipase C signaling, and CD28 signaling in T helper cells. These conserved pathways may be inherently crucial to the chicken's response to NDV in the trachea. The stress-activated protein kinase (SAPK)/Jun N-terminal protein kinase (JNK) signaling pathway was activated only in the Leghorns and was activated at all three time points. This pathway may be associated with the Leghorns' susceptibility to NDV (Fig. 6).

### Collagen downregulation at 2 dpi was identified only in the Fayoumi chickens.

**FIG 7:**
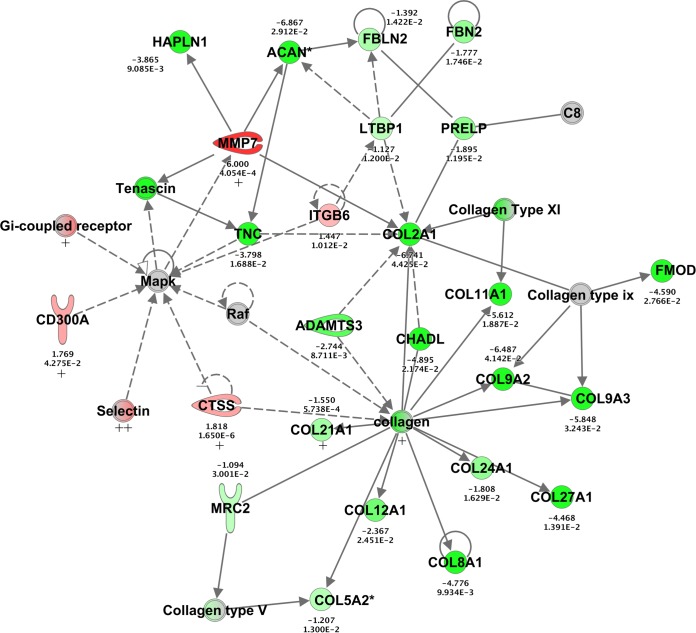
Collagen-related network generated from the challenged versus nonchallenged Fayoumi chickens at 2 dpi. For all genes, the absolute LFC was >1 and the FDR was <0.05. This network shows direct (solid lines) and indirect (dashed lines) relationships between genes that were either upregulated (red), downregulated (green), or not DE (gray) between the challenged and nonchallenged Fayoumis at 2 dpi. The LFC (top) and FDR (bottom) are shown for each gene. Of the 30 genes, 8 genes denoted with a plus sign were also DE in the Leghorns at this time. Qiagen IPA software was used to create this image.

The network shown in Fig. 7 shows the overwhelming downregulation of collagen, which was predicted by IPA to be associated with various diseases and functions, such as connective tissue disorders, dermatological diseases and conditions, and organismal injury and abnormalities. This network had 25 focus modules and had the second-highest score of the networks generated from this contrast. Of the 30 genes in this network, only 8 were also DE in the Leghorn chickens (Fig. 7). It does not appear that the downregulation of collagen was limited to a specific collagen type.

### Direct comparison of the Fayoumi and Leghorn chickens at 2 dpi gave more insight into the pathways and genes associated with resistance.

**FIG 8:**
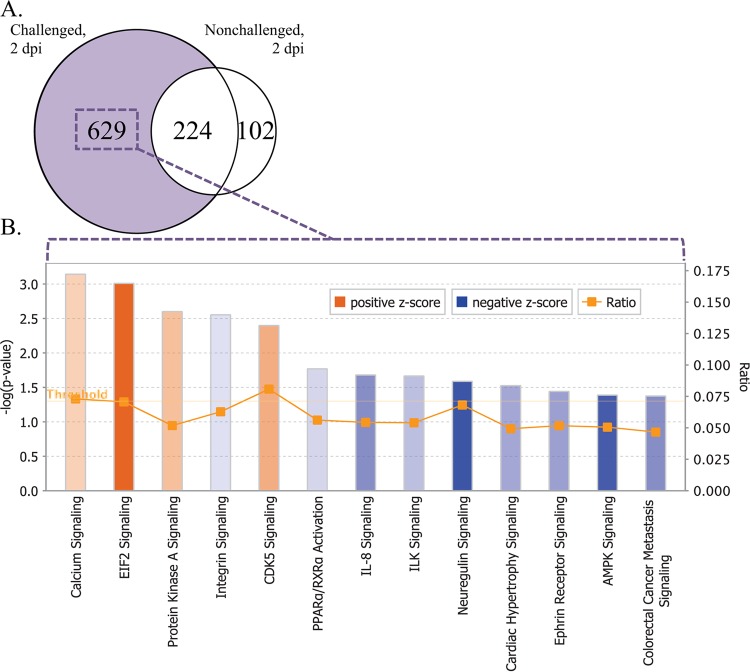
Contrasting the Fayoumi and Leghorn chickens at 2 dpi. (A) Venn diagram showing differentially expressed genes (FDR < 0.05) between the Fayoumis and Leghorns in the challenged group (left) and the nonchallenged group (right). The 629 genes (purple) that were uniquely identified to be DE in the challenged group were used for pathway analysis. (B) Top canonical pathways of the genes differentially expressed (FDR < 0.05) between the two lines at 2 dpi in the challenged birds. Canonical pathways [Z-score > 0.05, −log(*P* value) > 1.3] in orange are more likely to be activated in the Fayoumis, and those in blue are more likely to be activated in the Leghorns. The height of each bar corresponds to the −log(*P* value) associated with each pathway, and the lower that the transparency of each bar's fill is, the higher absolute value that the Z-score is. The ratio (orange line) represents the proportion of genes within the pathway that were DE. ILK, integrin-linked kinase; AMPK, AMP-activated protein kinase.

The Fayoumis and Leghorns had a high degree of similarity in the cell populations enriched at 2 dpi (Fig. 4), allowing for direct comparison between the two lines. The number of DEG between the Fayoumis and Leghorns was higher in the challenged birds than the nonchallenged birds (Fig. 8). Myosin- and troponin-related genes, as well as MHC class I genes, were the most upregulated genes in the Fayoumis relative to the Leghorns at this time. The eukaryotic translation initiation factor 2 (eIF2) signaling pathway had the highest positive Z-score (Fig. 8), suggesting strong activation of this pathway in the Fayoumi chickens relative to the Leghorn chickens.

### Examining the genes that were impacted by challenge and line offered potential candidate genes of interest.

A contrast was written to compare the interaction between challenge and line at each time point. At each time point, only DEG from the challenged versus nonchallenged contrast within each line were examined for the interaction (line · challenge). The majority of the genes with a false discovery rate (FDR) of <0.05 for the interaction were from 6 dpi, and nearly half of all genes were uncharacterized proteins (Table 2). Further investigation into the function of these uncharacterized proteins would be useful to provide more insight into the resistant and susceptible phenotypes of Fayoumi and Leghorn chickens.

**TABLE 2:** Genes whose expression levels were significantly affected by challenge and line

Ensembl transcript identifier	Gene name^*a*^	LFC^*b*^	FDR^*c*^	dpi^*d*^	Description^*a*^	Source	Accession no.
ENSGALT00000015621	*DMBT1*	3.09	0.00574	2	Uncharacterized protein	UniProtKB/TrEMBL	E1C1A5
ENSGALT00000034031		−5.82	0.00004	6	Uncharacterized protein	UniProtKB/TrEMBL	F1NSC8
ENSGALT00000009555		−4.73	0.00034	6	Uncharacterized protein	UniProtKB/TrEMBL	F1NC22
ENSGALT00000018840	*IGJ*	−4.63	0.00034	6	Immunoglobulin J polypeptide, linker protein for immunoglobulin alpha and mu polypeptide precursors	RefSeq peptide	NP_989594
ENSGALT00000034032		−5.11	0.00046	6	Uncharacterized protein	UniProtKB/TrEMBL	F1NSC7
ENSGALT00000009519		−4.69	0.00052	6	Ig lambda chain V-1 region	UniProtKB/Swiss-Prot	P04210
ENSGALT00000009564		−4.81	0.00052	6	Uncharacterized protein	UniProtKB/TrEMBL	F1NBX7
ENSGALT00000002609	*GABRA1*	4.48	0.00064	6	Gamma-aminobutyric acid receptor subunit alpha-1	UniProtKB/Swiss-Prot	P19150
ENSGALT00000008309	*STX3*	−2.86	0.00148	6	Syntaxin 3	HGNC^*e*^ symbol	11438
ENSGALT00000011371		−3.08	0.00351	6	Uncharacterized protein	UniProtKB/TrEMBL	F1NGI5
ENSGALT00000003541		−4.45	0.00416	6	Uncharacterized protein	UniProtKB/TrEMBL	H9KZ23
ENSGALT00000004568	*ENPP3*	−3.98	0.00705	6	Ectonucleotide pyrophosphatase/phosphodiesterase 3	HGNC symbol	3358
ENSGALT00000043739		4.41	0.01388	6	Uncharacterized protein	UniProtKB/TrEMBL	R4GFG6
ENSGALT00000025174	*DOK7*	2.11	0.01447	6	Docking protein 7	HGNC symbol	26594
ENSGALT00000002058	*TEKT3*	3.89	0.01473	6	Tektin 3	HGNC symbol	14293
ENSGALT00000022671		−1.45	0.01473	6	Uncharacterized protein	UniProtKB/TrEMBL	E1BUA6
ENSGALT00000034018		−4.12	0.01984	6	Uncharacterized protein	UniProtKB/TrEMBL	F1NSD3
ENSGALT00000040746	*TNFRSF13B*	−4.29	0.03047	6	Tumor necrosis factor receptor superfamily member 13B	RefSeq peptide	NP_001091006
ENSGALT00000038130	*HEPACAM2*	1.97	0.03127	6	HEPACAM family member 2	HGNC symbol	27364
ENSGALT00000037100	*CTNND2*	1.76	0.03708	6	Catenin delta 2	HGNC symbol	2516
ENSGALT00000043820	*MZB1*	−3.68	0.03827	6	Marginal zone B and B1 cell-specific protein	HGNC symbol	30125
ENSGALT00000006673		−1.88	0.04387	6	Uncharacterized protein	UniProtKB/TrEMBL	F1P1M1
ENSGALT00000036627		1.71	0.04387	6	cHz-cadherin precursor	RefSeq peptide	NP_001001754
ENSGALT00000012349	*MEGF11*	1.51	0.04440	6	Multiple EGF-like-domains 11	HGNC symbol	29635
ENSGALT00000027128	*SULT1C*	−1.39	0.04901	6	Sulfotransferase 1C1	RefSeq peptide	NP_989932
ENSGALT00000031381		1.66	0.04901	6	Uncharacterized protein	UniProtKB/TrEMBL	E1C402
ENSGALT00000009564		−4.86	0.00638	10	Uncharacterized protein	UniProtKB/TrEMBL	F1NBX7
ENSGALT00000043908		3.33	0.00638	10	Uncharacterized protein	UniProtKB/TrEMBL	R4GM39
ENSGALT00000030326	*NYX*	4.54	0.03834	10	Nyctalopin	HGNC symbol	HGNC:8082
ENSGALT00000038746	*HDC*	2.67	0.03834	10	Histidine decarboxylase	HGNC symbol	4855

a Ensembl BioMart software was used to obtain the gene name and description from the transcript identifier.

b LFC, log_2_ fold change.

c FDR, false discovery rate.

d dpi, days postinfection.

e HGNC, Human Gene Nomenclature Committee.

### The RNA-seq results were validated with an independent test.

**FIG 9:**
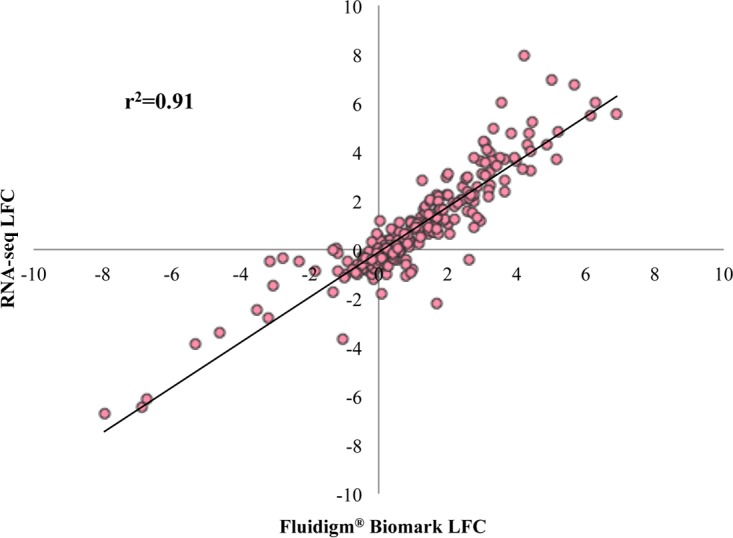
RNA-seq results validated using the Fluidigm Biomark HD system. This scatter plot shows the agreement between the LFC measured by RNA-seq and the LFC measured by the Fluidigm Biomark HD system. The LFCs were generated from the six major contrasts for the challenged and the nonchallenged birds at each time and in each line for 43 genes in total. Each dot represents the LFC of a gene in a particular contrast; there are 258 points in total. The pairwise correlation between the two methods was 0.91. H6PD was the housekeeping gene used for normalization of the data obtained with the Fluidigm Biomark HD system. See Table 3 for primer information.

The Fluidigm Biomark HD system was used as a method of high-throughput quantitative PCR to serve as validation for the transcriptome sequencing (RNA-seq) results. The expression of 44 genes (Table 3) was analyzed. Figure 9 shows a correlation of 0.91 between the two methods across all six major contrasts. The high correlation serves as a validation of the RNA-seq technology applied in this study.

**TABLE 3:** Primers used for validation of RNA-seq data using the Fluidigm Biomark HD high-throughput qPCR system

Associated gene name	Sequence		Ensembl transcript identifier
	Forward primer	Reverse primer	
*ACKR2*	GACATCCAGCTCTCAGAGACA	CACGTGTTGGTGATGCTCAA	ENSGALT00000008506
*AJAP1*	GCCTGGAGATTACAAAGCAACC	AGACACAAAGGCCACAGGAA	ENSGALT00000044613
*APOA1*	CCCTCGCTGTGCTCTTCC	GCCTTCACCGTCTCCAGGTA	ENSGALT00000011524
*CASP7*^*a*^	ATGACCGAAGCTGTGAGGATA	GGCAAAACAAGCAGCATCAC	ENSGALT00000014519
*CASP9*^*a*^	TTTCAGGTCCCTGTGCTTCC	TTCCGCAGCTCCACATCAA	ENSGALT00000002085
*CCR8*	TCTGTGAGGCTTTCGGCAAA	TCCGTGTTGCGTCTGTTGAA	ENSGALT00000019499
*CD4*	AGTGGAACCTGGATGTGTCA	TTTCCAAGCGTTCCTTCTCAAA	ENSGALT00000037104
*CD40*^*a*^	AGCCTGGTGATGCTGTGAA	CTCACAGGGTGTGCAGACA	ENSGALT00000039105
*CMPK2*	AGGCTGAACTGGAAGCTAACA	CTTGGCACGCAGGATTCAC	ENSGALT00000043751
*COL2A1_s5*	GGTCCTTCCGGCTTCCA	CAGGAACACCCTGGTCTCC	ENSGALT00000035835
*COL5A2*	TGGGAACCACCTGACACAAA	GAGCCAAACGTCAGCTTCAA	ENSGALT00000042826
*COL9A2*	CCAAGGCTTGCCAGGAGTCA	CCTTTGGGACCGGTCTTTCC	ENSGALT00000043674
*CREB3L1*	ATGCTGAAACCAGCGAGGTA	AACAGCTGCCTGCTCACTTA	ENSGALT00000013673
*CRISP3*	AGGCGAGACTTCCAATCTTCC	GACAGCACAGCTGCAAGAC	ENSGALT00000026918
*H6PD*^*a*^	ATGTACCGGGTGGACCACTA	AACTGACGGTTCTGATCTCGAAA	ENSGALT00000003926
*HCK*	ACTGGAAACAGAGCTCAGAGAC	TCTGCAGGAAGCAGAGAAACA	ENSGALT00000010538
*IL-18*^*a*^	CGTGGCAGCTTTTGAAGATGTA	CTGAATGCAACAGGCATCCC	ENSGALT00000012787
*IL1B*^*a*^	TGCTTCGTGCTGGAGTCAC	GGCATCTGCCCAGTTCCA	ENSGALT00000000738
*IL-6*^*a*^	AACGTCGAGTCTCTGTGCTA	AGTCTGGGATGACCACTTCA	ENSGALT00000017759
*IL-8*^*a*^	CCCCACTGCAAGAATGTTGAAA	GTGCCTTTACGATCAGCTGTAC	ENSGALT00000042745
*ITK*	TGGAACAAGTGCCAGACCAA	AAGTCGCTATGGCTTTGGCTA	ENSGALT00000006130
*LAG3*	CAGAACCAGAGCAGCAGAAAC	CAACAGTGACAGCACAGCAA	ENSGALT00000023369
*LITAF*^*a*^	ATCGTGACACGTCTCTGCTA	AGCATCAACGCAAAAGGGAA	ENSGALT00000005095
*MAPK8IP3*^*a*^	GCCAAAGCCAAAATGGAAACC	GTCGAGCAACAATCGCTTCA	ENSGALT00000002721
*MHCI-likeY*	GCCGGAACGCTACAACAAA	TCCAGGATGTCACAGCCAAA	ENSGALT00000009998
*MMP7*	CGGGACAGGCAGACATCA	TGTGCCACCTCTTCCATCAA	ENSGALT00000027770
*MOV10*	CAAGAAGCTGTTGAAGCCCTAC	AGGAAAGTTGGCCGATACCA	ENSGALT00000002368
*Mx*	CTGTTGCGATGCTGAACAAA	TTAGCAAAACGCTTGTTAGCC	ENSGALT00000025999
*MYO7A*	TGAGGCAGGAATCCTTAGGAAC	ACTGCCACCAGTATTGAACCA	ENSGALT00000001046
*NLRC5*^*a*^	TTTGCTGCTGCGCTTTCA	TGTGATGCTTCCACCTGTCA	ENSGALT00000001908
*PRKCB*	ATGTGGTACAGATGCCAACCA	AGCCCCAAACAGAACGGTAA	ENSGALT00000009676
*RHPN2*	TGACCGCACATACCGTAACA	AAGAGCTGTCCTGAACCAACA	ENSGALT00000007686
*RPL4*^*a*^	TTCTGCCTTGGCAGCATCA	AGGAAGTTCTGGGATCTCCTCA	ENSGALT00000012495
*SDHA*^*a*^	CGTGATCTGGCTCATCTAAAGAC	TTGCAGTTCAAGGGTCTCCA	ENSGALT00000021508
*SERPINB2*^*a*^	AAATCACCGTGCCTGTTTCC	GTGCTTTAAACCCAGCATGGATA	ENSGALT00000020982
*SLC16A6*	TCACAGCAGTGGCATCTACA	CAGCCAATCTGCTCCTTCAAA	ENSGALT00000006617
*SPON2*	TCACACCCAAGGTGGGAAAA	ACTCTGTGCTCCCTGCAAA	ENSGALT00000045656
*TGFB3*^*a*^	GGGCCCTGGATACCAACTAC	GGTCCTGTCGGAAGTCAATGTA	ENSGALT00000037707
*TLR4*^*a*^	CCTGCTGGCAGGATGCA	TGTTCTGTCCTGTGCATCTGAA	ENSGALT00000011333
*TRAF6*^*a*^	TGCCCCAGTACCATGCTTTTA	GTGTCGTGCCAGTTCATTCC	ENSGALT00000012888
*TRAT1*	CGTAGCACAGCACAGAAACA	ATTGTCCCCTGCTTCACTCA	ENSGALT00000036882
*WNT4*^*a*^	AGCAAAGGGGCATCTTCCAA	TCCACCCGCATGTTGTTCA	ENSGALT00000007645
Worthington	TTCACAGGCACCTACATCACA	GGTGTTTTGTGTCCCATGCA	ENSGALT00000003855
*ZNFX1*	TGCTGAAGTCTGCTGCTGTA	AACATTGGCCGTTCACTGAC	ENSGALT00000007763

a The primers were used/analyzed previously (62).

## DISCUSSION

Examining changes in the transcriptome of the tracheal epithelia between challenged and nonchallenged birds offered valuable insight into the host response to NDV. This study predicted the enrichment of immune cells in the trachea in challenged birds of both lines at all times. One pathway that was instrumental in leukocyte migration was leukocyte extravasation signaling. On the basis of the expression of genes within this pathway, IPA predicted the activation of actin cytoskeleton contraction and cell mobility. A previous study examining gene expression levels in the lung after challenge with AIV in the same experimental lines showed an upregulation of actin filament-based movement in the Fayoumis (29). Actin cytoskeleton activation is crucial for cell migration, as leukocytes use actin polymerization and actomyosin contraction for movement. Notably, paramyxoviruses like NDV also require actin to replicate efficiently (32, 33). If the migrating cells become infected with the virus, a conundrum may be created for the host. In one study, there was a downregulation of actin-related genes after challenge with NDV *in vitro* (34). Due to challenge with infectious bronchitis virus (IBV), the proteins involved in cytoskeleton organization differed in expression in the trachea depending on IBV virulence (35). The host must find a way to balance the costs and benefits of cytoskeleton upregulation with defense against viral infection. The regulation of cytoskeleton- and actin-related genes may be time dependent and may be a critical target for future study.

Host-pathogen interactions at the site of infection can determine the severity of both the pathology and the overall infection. The use of two unique inbred lines that differ in their relative resistance offered a valuable tool for comparison. Fayoumis had significantly less detectable virus at 2 dpi (measured by RNA-seq) and 6 dpi (measured by qPCR), which may result in faster viral clearing and which, overall, supports their relatively resistant phenotype compared to the phenotype of the Leghorns. A decreased viral load corresponds to less viral shedding and, therefore, fewer viral particles in the environment and a lower transmission potential (36). In a separate study with the same two genetic lines, although all birds succumbed to infection with velogenic NDV, the mean survival time was longer for the Fayoumis than the Leghorns at all four doses tested and was significantly longer at the two higher doses (P. Miller and C. Afonso, SEPRL, Athens, GA, personal communication, 2016).

As the time postinfection increased, the numbers of DEG between challenged and nonchallenged birds decreased, suggesting that the chickens were recovering. The Fayoumis had many fewer DEG at 6 dpi than the Leghorns, which may be further evidence that the Fayoumis recovered more quickly. Although most pathways had similar predicted levels of activation or inhibition in the two lines, the lines often differed in gene expression levels within the pathway (Fig. 5). The current study identified several genes whose expression levels were impacted significantly by both line and challenge (Table 2), and some of these overlapped those from a previous study. In the lung, *TNFRSF13B* and *IGJ* were DE between the Fayoumi and Leghorn chickens, and the Ig lambda chain V-1 region was DE in both lines after challenge with AIV (29). These genes may be of particular importance in the host response to both AIV and NDV.

The wide range of pathogens (parasites, bacteria, viruses) to which the Fayoumis have shown relative resistance suggests that the mechanism(s) for their resistance may function early in the immune response (26, 27, 29). Downregulation of collagen at 2 dpi in the challenged Fayoumis could be partially responsible for their resistant phenotype. One study found lower levels of expression of collagen mRNA in the trachea of resistant chickens (noninbred Cornell chickens) than susceptible chickens (inbred White Leghorn chickens) after challenge with IBV (37). The downregulation of collagen may impact apoptosis (38), immune cell migration (39–41), and T-cell activation (42–46); impacting these processes would likely impact the virus and its pathogenesis.

A direct comparison of the challenged Fayoumis and Leghorns at 2 dpi also gave insightful results. Integrin signaling was more highly activated in the Leghorns than the Fayoumis (Fig. 8). This may be directly related to the downregulation of collagen and its relationship with integrin (38). Laminin, another key extracellular matrix protein (39), was also upregulated in the Leghorns relative to the Fayoumis. The impact of collagen was shown in two separate contrasts. The downregulation of collagen between challenged and nonchallenged Fayoumis and the activation of collagen-related pathways in the Leghorns relative to the Fayoumis give more confidence in the importance of collagen at 2 dpi.

The activation of the protein kinase R (PKR)/eIF2 signaling cascade is known to inhibit NDV replication (47), likely due to the actions of the antiviral protein PKR. This pathway, involved in mRNA translation, was more upregulated in the Fayoumis than the Leghorns (Fig. 8). This finding was in agreement with the Fayoumis' resistant phenotype and may be related to the lower viral load seen at 6 dpi. The phosphorylation of eIF2α results in apoptosis due to a global termination of transcription (48). Apoptosis-related pathways were activated, including T lymphocyte apoptosis, p53 signaling, retinoic acid-mediated apoptosis signaling, and apoptosis signaling (Fig. 6). The eIF2 signaling pathway was also identified to be differentially methylated between chickens that were either vaccinated or not for infectious laryngotracheitis (48). It is clear that this pathway is critical in the host defense against multiple pathogens.

In a previous study, chicken stem cells underwent rounds of selection with high titers of the La Sota strain; the levels of expression of these selected cells led to increased levels of apoptosis and decreased neuroactive ligand-receptor interaction compared to those in the unselected cells (49). The Fayoumis showed higher levels of activation of apoptosis signaling at 2 dpi (Fig. 6), and when the lines were directly compared, the Leghorns showed more activation of the neuregulin signaling pathway at 2 dpi (Fig. 8). The overlap in these pathways suggests that increased levels of apoptosis and decreased neurologically related pathways may be important mechanisms of host resistance to La Sota.

The trachea transcriptome exposed potential key mechanisms for the host defense against NDV. As expected, there appeared to be a large increase in immune cell infiltration in the trachea in both lines at all times examined. Although shared mechanisms were seen in both the Fayoumis and Leghorns, the lines showed unique responses as well. This study has demonstrated the Fayoumis' classification as being relatively resistant to NDV compared to the Leghorns, as determined by viral load and viral sequence counts and suggested by antibody titers and DEG numbers. This study identified possible mechanisms for the Fayoumis' resistance, including the downregulation of collagen and the increased activation of eIF2 signaling, which can be used to improve vaccine development and highlight genes for which beneficial genetic variance can be used to inform breeding decisions. Cytokine expression levels are time, tissue, and strain dependent (11–13). It is critical that future breeding decisions be made from data from studies with multiple tissues, host genetic backgrounds, NDV strains, and time points. The current study is a first step in understanding and identifying possible mechanisms of resistance to NDV. Future studies to examine the gene expression of Fayoumis and Leghorns challenged with velogenic NDV are necessary to better determine the applicability of the results presented here.

## MATERIALS AND METHODS

### Ethics statement and animals used.

The methods used in the present study were approved by the Iowa State University IACUC (log number 1-13-7490-G). Fayoumi (M 15.2) and Leghorn (GHs 6) chicken lines from Iowa State University Poultry Farm (Ames, IA) were used in this study. The Fayoumis and Leghorns each have an inbreeding coefficient of >99.9%, as determined by microsatellites (50), and 99.95%, as determined by resequencing (51). The Fayoumi line originated from the Fayoum region of Egypt, whereas the Leghorn line is representative of chickens from the U.S. egg-laying industry of the 1950s. The chickens were given *ad libitum* access to water and feed throughout the study. Supplemental heat at 35°C was provided to the birds starting on the day of hatch, and the temperature was stepped down 2 to 3°C every few days until it reached 24°C when the chicks were 29 days of age and remained at 24°C for the remainder of the study. The light cycle began at 23 h of light and 1 h of dark on the day of hatch, and the number of hours of light was gradually reduced until it reached 13.5 h of light when the chicks were 24 days of age and remained at that amount for the remainder of the study.

### Virus.

A live attenuated Newcastle disease vaccine, the type B1 La Sota lentogenic strain, was propagated once in 10-day-old embryonated specific-pathogen-free (SPF) chicken eggs inoculated via the allantoic sac (52). The allantoic fluid was harvested after 2 days, tested for virus presence by determination of hemagglutinating activity (HA), and centrifuged at 8,000 rpm for 30 min to clear the debris. Virus titration was performed in SPF embryonated eggs as previously described (53). The final titer of the undiluted virus suspension was 10^8^ 50% embryo infectious dose (EID_50_) per ml. The virus suspension was aliquoted and stored at −80°C until its use.

### Experimental design.

At the time of hatch, straight run chickens of both lines were randomly assigned to one of four rooms in a biosafety level II facility. At 21 days of age, half of the chickens (two rooms, *n* = 49) were inoculated with 200 μl of 10^7^ EID_50_ of La Sota NDV, with 50 μl being inoculated into each of the eyes and nostrils. The nonchallenged group (2 rooms, *n* = 40) was given 200 μl phosphate-buffered saline (PBS), via the same route, as a mock infection. Chickens that received the La Sota virus are referred to throughout this article as “challenged,” and those that received the mock infection are referred to as “nonchallenged.” To collect lachrymal fluid prechallenge (*n* = 89) and at times of 2 (*n* = 89) and 6 (*n* = 62) dpi, fine, crystalline sodium chloride was placed on the eye of each chick to collect lachrymal fluid with a pipette. To quantify antibody levels, venous blood samples were collected prechallenge and at 10 dpi. At 2, 6, and 10 dpi, 24, 30, and 35 chicks, respectively, were euthanized with sodium pentobarbital solution for tissue harvest. The entire trachea was harvested from all of the challenged and nonchallenged birds sacrificed at each dpi and placed into RNAlater solution (Thermo Fisher Scientific, Waltham, MA) for short-term storage. Within a week, the sheet of epithelial cells from each trachea was carefully removed by peeling it from the interior surface of the trachea with forceps. This epithelial cell lining was placed into a −80°C freezer for long-term storage, and the remaining tissue was discarded.

### NDV antibody.

Blood samples collected prechallenge and at 10 dpi were centrifuged at 15 rpm for 5 min to collect the serum supernatant. Serum NDV antibody levels were measured using an Idexx NDV ELISA for chickens (Idexx Laboratories, Inc., Westbrook, ME). The sample-to-positive (S/P) ratio was calculated by subtracting the negative-control value from the average absorption value for each sample and dividing that value by the positive-control value minus the negative-control value. The positive and negative controls were supplied with the Idexx kit. The S/P ratio was calculated from the average absorbance value for each sample, which was run in duplicate. A standard least-squares, effect leverage analysis in JMP (JMP Group Inc., San Francisco, CA) including the main effects of line, treatment, and line · treatment was used; a Student *t* test generated the connecting letters report. Both tests are parametric.

### Viral load.

Viral RNA was isolated from the chicken lachrymal fluid collected prechallenge and at 2 and 6 dpi using a MagMAX-96 viral RNA isolation kit (Life Technologies, Carlsbad, CA) and was quantified using an LSI VetMAX Newcastle disease virus real-time PCR kit (Life Technologies, Carlsbad, CA) on an MJ Research Opticon2 qPCR machine (Bio-Rad, Hercules, CA). The primers were directed against the matrix protein (M) gene, and dilutions of inoculum virus were used as standards. The log copy number per 1 μl of isolated viral RNA was calculated for the mean for each sample, which was run in duplicate. Using JMP statistical software (JMP Group Inc., San Francisco, CA), a standard least-squares, effect leverage test including the main effects of line, dpi, and line · dpi was performed to determine significance, and a Student *t* test was utilized to generate a connecting letters report. The log copy number of the NDV genome does not show the number of infectious particles.

### RNA isolation and cDNA library construction.

RNA was isolated from the tracheal epithelial cells using an RNAqueous kit (Thermo Fisher Scientific, Waltham, MA), and samples were then treated with DNase using a DNA-free kit (Thermo Fisher Scientific, Waltham, MA). The quality of all samples was validated (RNA quality number > 8.0) using the Fragment Analyzer (Advanced Analytical Technologies, Ankeny, IA). A 500-ng input for each sample was utilized to construct the cDNA library using the high-throughput protocol in the TruSeq RNA sample preparation guide (v2; Illumina, San Diego, CA). Following cDNA library validation using the Fragment Analyzer (Advanced Analytical Technologies, Ankeny, IA), samples were sequenced on a HiSeq2500 platform (Illumina, San Diego, CA) for 100-bp, single-end reads (DNA Facility, Iowa State University, Ames, IA).

### RNA-seq design.

This study included 3 main factors: treatment (challenged, nonchallenged), line (Leghorn, Fayoumi), and time (2, 6, 10 dpi), resulting in 12 treatment groups. Each treatment group was represented by 4 biological replicates, except for the Leghorns at 2 dpi, for which there were 3 nonchallenged and 5 challenged chickens included in the analysis. Equal numbers of males and females were used for each treatment group. Samples from birds in the 12 treatment groups were balanced across four lanes of the flow cell and randomly assigned an index.

### Data analyses.

The Discovery Environment of iPlant Collaborative (54) was utilized for data processing. First, the FastQC program (http://www.bioinformatics.babraham.ac.uk/projects/fastqc/) was utilized for an initial quality check and assessment of all sequence data. The Illumina TruSeq adapter was recognized as an overrepresented sequence in some samples. For consistency, the adapter sequence and the individual multiplexing index were removed from all samples using the FASTX Clipper program. In addition, FASTX removed any sequence less than 30 bp in length and filtered each read such that 80% of all base pairs within a read had a Phred quality score of 30 or higher. The remaining high-quality reads were used as input for the TopHat2 aligner (55) for alignment to the Galgal4 reference genome (assembly GCA_000002315.2). The Galgal4 (assembly GCA_000002315.2) and GTF files were downloaded from the Ensembl genome browser. In TopHat2 (55), default settings were used, except the minimum isoform fraction was lowered to 0.10 (the default is 0.15) to identify more alternatively spliced transcripts. The HTSeq library (56) was used to count the reads aligned to each transcript (the intersection-nonempty mode was used). Statistical analysis of the count data was performed using the generalized linear model option in the edgeR package (57). The main effects of line, treatment, and time were included in the model. The contrasts comparing the challenged and nonchallenged birds within each line at each time are referred to as the six major contrasts (Fayoumi, 2 dpi, challenged versus nonchallenged; Leghorn, 2 dpi, challenged versus nonchallenged; Fayoumi, 6 dpi, challenged versus nonchallenged; Leghorn, 6 dpi, challenged versus nonchallenged; Fayoumi, 10 dpi, challenged versus nonchallenged; Leghorn, 10 dpi, challenged versus nonchallenged).

The differentially expressed genes (DEG; FDR < 0.05) identified in either chicken line when comparing challenged to nonchallenged included 1,688, 965, and 180 DEG at 2, 6, and 10 dpi, respectively (Fig. 3). For these genes, contrasts were written to determine the significance of the interaction between line and challenge at each time. The *P* values generated from the interaction contrast were subjected to a Benjamini and Hochberg multiple testing correction (58).

### NDV sequence discovery.

The NDV La Sota sequence (GenBank accession number JF950510.1) was downloaded from NCBI and used to make a GFF file. Using the Burrows-Wheeler alignment (BWA) (59) under default settings, the unmapped reads were aligned to the NDV genome (GenBank accession number JF950510.1). The HTSeq library (56) was used to count the number of reads that aligned to each viral gene. The cpm was calculated by taking the number of reads that aligned to each viral gene, dividing by the total number of unmapped reads, and multiplying by 1 million. There was no statistically significant difference between the number of unmapped reads in the challenged Leghorns and Fayoumis at 2 dpi (*P* = 0.4). The data were imported into JMP statistical software (JMP Group Inc., San Francisco, CA) and analyzed with a least-squares analysis and Student's *t* test to generate the connecting letters report.

### Gene expression analysis.

The trachea is a heterogeneous tissue composed of multiple cell populations that likely change under different treatment conditions. To determine cell type enrichment, the transcript identifiers of the DEG (FDR < 0.05) for each of the six major contrasts were converted into their associated gene names using BioMart software (http://uswest.ensembl.org/biomart/martview/). The six gene lists were then input into the CTen database (http://www.influenza-x.org/~jshoemaker/cten/) (60). The CTen database is based on human and mouse data and has previously been used for chickens (61).

For pathway analysis, Qiagen's Ingenuity Pathway Analysis (IPA; Qiagen, Redwood City, CA) was used. The identifiers of the transcripts unrecognized by IPA were converted to a recognized identifier using BioMart software (http://uswest.ensembl.org/biomart/martview/) to ensure that the maximum number of transcripts was included for pathway analysis. The genes used for pathway analysis had an FDR of <0.05 and an absolute log_2_ fold change (LFC) of >1, as estimated by the edgeR package.

### Fluidigm Biomark HD system.

To validate the RNA-seq results, the Fluidigm Biomark HD system (Fluidigm, South San Francisco, CA) was used as a method of high-throughput qPCR. The input RNA (25 ng/μl) came from the same isolate used for cDNA library construction. The 48 RNA samples were converted into cDNA, preamplified for 12 cycles with all 48 primer pairs (Table 3), treated with exonuclease, diluted 10-fold, and analyzed on the 48.48 integrated fluidic circuit (IFC) for a total of 2,304 sample · gene tests. The genes were chosen to represent the range of log fold changes; not all genes were DE. Ribosomal protein L4 (RPL4) and hexose-6-phosphate dehydrogenase (H6PD) were two housekeeping genes with a high raw cycle threshold (*C_T_*) value correlation (*r* = 0.9). Since its *C_T_* values were closer to the average *C_T_* values for all primers and samples, H6PD was used as the housekeeping gene for the analysis. Data were filtered by Fluidigm quality assessment and melting curve consistency. If, after these filtering steps, primer pairs did not have more than 28 samples remaining, all measurements for that gene were excluded. A total of 9.5% of the data, including those for four genes (*MZB1*, *5_8S_rRNA*, *UbI*, and *GAL2*), were excluded from the analysis. Table 3 shows the genes and primers analyzed in this study.

For all six major contrasts, the LFC in expression for each gene was calculated by the −2^−ΔΔ*CT*^ method. The correlation between the RNA-seq-generated LFC and the Fluidigm Biomark HD system-generated LFC was determined with a pairwise correlation across all genes and all six major contrasts for 258 pairwise comparisons in total.

### Accession number(s).

Gene expression data were deposited in ArrayExpress, EMBL/EBI, under accession number E-MTAB-5431.
